# Simulating crisis triage: a methodological framework for evaluating ventilator allocation under crisis standards of care

**DOI:** 10.1186/s12874-026-02878-1

**Published:** 2026-05-26

**Authors:** B. Corbett Walsh, Jianan Zhu, Yang Feng, Rebecca A. Betensky, Deepak Pradhan

**Affiliations:** 1 Division of Pulmonary, Critical Care, and Sleep Medicine, Department of Medicine, University of Los Angeles David Geffen School of Medicine, Los Angeles, CA USA; 2 Section of Palliative Medicine, Department of Medicine, University of Los Angeles David Geffen School of Medicine, Los Angeles, CA USA; 3 Department of Biostatistics, New York University School of Global Public Health, New York, NY USA; 4 Division of Pulmonary, Critical Care, and Sleep Medicine, Department of Medicine, New York University Grossman School of Medicine, New York, NY USA; 5 New York University Langone Health, New York, NY USA; 6 Bellevue Hospital Center, NYC Health and Hospitals, New York, NY USA; 7 UCLA David Geffen School of Medicine, 10833 Le Conte Ave, Los Angeles, CA 90095 USA

**Keywords:** COVID-19, Decision Making, Ethics, Ventilator, Resource Allocation, Crisis standards of care, Ventilator allocation, Triage protocols, Simulation modeling, Computational simulation

## Abstract

**Background:**

Crisis Standards of Care (CSC) may require rationing of life-sustaining resources, such as mechanical ventilation, during public health emergencies. Simulation modeling offers a scalable, transparent method to evaluate triage frameworks before implementation. While ventilator triage frameworks vary in their use of exclusion criteria, comorbidity adjustments, and reassessment frequency, few have been rigorously compared using real-world data under realistic surge conditions. Robust platforms that simulate both front-end (initiation) and back-end (reassessment or reallocation) triage are critical for evaluating clinical, operational, and ethical performance.

**Methods:**

We developed a computational simulation platform using retrospective real-world data from intubated adults during the Spring 2020 COVID-19 surge across a large New York City health system. The simulated surge cohort included all patients mechanically ventilated between March 1 and June 30, 2020. A crisis cohort was defined as those patients receiving ventilation once 95% of the health system’s pre-pandemic ventilator supply was in use. Eight CSC strategies were evaluated, including policies from New York, Pennsylvania, Maryland, Canada, two academic frameworks, a lottery-based system, and first-come first-served. Strategies varied in their use of exclusion criteria, comorbidity modifiers, reassessment intervals, and prioritization for special populations. Daily ICU census and ventilator availability were used to simulate resource strain and drive triage decision-making. Patients simulated for ventilator rationing were simulated to expire. Manual abstraction of comorbidities and structured rules for imputing missing SOFA subscores were applied uniformly.

**Results:**

The platform simulated 10,000 iterations per strategy and included 2,365 intubated patients. Final analyses will be published separately.

**Conclusion:**

This platform provides a scalable, reproducible framework for evaluating ventilator triage strategies under pandemic-like conditions. By integrating both initial triage and serial reassessment (front- and back-end) logic, operational constraints, and clinical trajectories, it enables detailed comparisons of survival, resource utilization, and prognostic accuracy. The simulation also supports ethical evaluation by testing the practical impact of exclusion and comorbidity-based criteria. Such models can assist governments, health systems, and public health agencies in designing triage protocols that are evidence-informed, ethically defensible, and operationally feasible. This work demonstrates how computational modeling can strengthen health system preparedness and support public trust.

**Supplementary Information:**

The online version contains supplementary material available at 10.1186/s12874-026-02878-1.

## Introduction

The COVID-19 pandemic exposed the fragility of healthcare systems operating under extreme resource strain. In catastrophic scenarios, crisis standards of care (CSC) may be activated to allocate life-sustaining resources, such as mechanical ventilators, based on predicted survival rather than clinical need alone [1]. These strategies aim to maximize lives saved, but differ substantially in design and implementation, raising profound ethical and legal concerns [2]. Understanding how various CSC strategies perform under dynamic, prolonged, and real-world conditions remains a critical priority for future pandemic preparedness and equitable public health response.

While all CSC strategies emphasize short-term prognostication–almost ubiquitously via the Sequential Organ Failure Assessment (SOFA) score, a summative 0–24 score of organ dysfunction across six organ systems–most apply this score in a static manner, using only the value from the day of triage [3]. Others adopt dynamic prognostication, comparing a patient’s current SOFA score to a prior SOFA score to assess clinical trajectory [4]. In addition, many CSC strategies incorporate triage modifiers, such as admission diagnosis (e.g., cardiac arrest, trauma), comorbidities (e.g., advanced malignancy, end-stage liver disease), age, pregnancy, occupation, or markers of social vulnerability [5–11]. These modifiers may temporarily exempt patients from triage, increase or decrease their overall triage prioritization, or functionally exclude them by assigning them to the lowest triage category. The use of such modifiers remains controversial, as they may disadvantage individuals with chronic conditions, exacerbate racial and socioeconomic disparities, or compromise allocation efficiency due to unreliable prognostication [12, 13]. In response to these concerns, some have advocated for unweighted lottery systems, which emphasize moral equality, distributive equity (by providing equal opportunity to all eligible recipients), and procedural fairness (through impartial and transparent allocation decision-making). Alternatively, a first-come first-served strategy–common practice in conventional or contingency standards of care–is simple to implement but agnostic to survival potential, and may penalize disadvantaged populations with lower health system access due to socioeconomic barriers, limited access to accurate pandemic information, transportation limitations, or distrust of the healthcare system [14]. 

Resource rationing under CSC includes two distinct processes: front-end triage, determining initial allocation decisions (whether a patient receives a ventilator), and back-end triage, involving periodic reassessments to decide if a ventilator therapy should be continued or withdrawn to reallocate the resource to others. To address existing gaps in understanding these dual processes, we developed a comprehensive simulation platform that models multiple CSC strategies for ventilator rationing in a dynamic, time-stepped manner, using real-world data during the initial COVID-19 surge from a large academic health system in New York City – the epicenter of the U.S. pandemic. Because CSC policies cannot be ethically evaluated through prospective real-world experimentation, simulation provides a method to compare competing triage frameworks under shared surge conditions [15]. Such head-to-head modeling can generate counterfactual evidence about survival, resource use, and potential inequities, thereby informing health system preparedness and policy development. The platform incorporates daily reassessments, realistic patient throughput, allocation and reallocation decisions, and reflects the operational complexity of prolonged high-strain environments. This platform builds upon our previous work scrutinizing a single strategy [4]. Strategies simulated include a non-weighted lottery, first-come first-served, and protocols developed by New York State [14], the University of California [16], and authorship groups from Maryland [11], Pennsylvania [9, 17], and Saskatchewan, Canada [10]. The primary aim of the study was to identify which strategy optimizes survival within the crisis cohort. Secondary aims included assessing ventilator rationing efficiency, and examining how various strategies and triage modifiers, particularly comorbidities, might exacerbate existing social disparities.

## Methods

### Study population and setting

We retrospectively reviewed adult patient records from a single academic hospital system in New York City during the initial COVID-19 surge, from 3/1/2020-7/1/2020. Patients were included if they received invasive mechanical ventilation. The health system had 263 pre-pandemic ventilators, excluding anesthesia machines [18]. In this simulation platform, a crisis period requiring ventilator triage was defined as the point at which ventilator demand exceeded 95% of pre-pandemic ventilator capacity (250 ventilators) [19], making an operational tipping point beyond which sustainable care delivery was no longer feasible and rationing became necessary. This period continued until two consecutive days of ventilator surplus occurred.

### Ventilator use estimation and simulation timing

Real-world ventilator use was queried from the electronic health record. However, because ventilator documentation was occasionally incomplete, undocumented ventilator-days were amended as continuous ventilator use only when they fit predefined short-gap patterns bounded by documented ventilator use (Table 1). These one- or two-day undocumented intervals were treated as suspected documentation gaps rather than true interruptions in invasive mechanical ventilation where a ventilator could be theoretically assigned to another patient. This amendment accounted for 2.2% (522/24,138) of all patient-ventilator-days. Patients later requiring ventilation were treated as reintubations and were reconsidered for ventilator allocation.

**Table 1:** Amended data set for continuous ventilator use

	Day_x_	Day_x+1_	Day_x+2_	Day_x+3_	Day_x+4_	Day_x+5_	Day_x+6_	Day_x+7_	#
Scenario 1	D	D	D	U	U	D	D	D	90
Scenario 2	D	D	U	U	D				32
Scenario 3	D	U	U	D					12
Scenario 4	D	D	D	U	D	D	D		167
Scenario 5	D	D	U	D	D				52
Scenario 6	D	U	D						169
Total (# / all patient-ventilator-days)									522/24,138 (2.2%)

D: Documented ventilator use

U: Undocumented ventilator use subsequently amended as ventilator use for analysis

All patient events (intubation, extubation, and death) were simulated using real dates but adjusted times, for all simulation models except the First-Come First-Served model, which preserved original intubation times. Except for the FCFS model, all strategies used a standardized daily 10:00 AM triage assessment, at which initial ventilator allocation and ventilator rationing decisions were simulated to occur. FCFS was modeled separately using original intubation times to preserve the temporal ordering of access, with newly available ventilators assigned beginning at midnight. Patients who had their ventilator rationed (either taken away or not provided) were simulated to expire as simulated in other comparable studies [4, 20–22]. This simplifying assumption – though clinically grounded – may overestimate mortality in rare cases. To explore this, we conducted a sensitivity analysis in which patients selected for ventilator rationing who had survived in the real world were still removed from the simulation, but were modeled to expire in only 70% or 90% of simulations rather than 100%. Patients who were extubated, died, or were placed on extracorporeal membrane oxygenation (ECMO) exited the simulation at 11:00 PM. The 11:00 PM exit time was selected as a standardized end-of-day modeling assumption within the daily time-step framework, rather than to reflect the precise timing of real-world clinical events. Accordingly, ventilators that became newly available after the 10:00 AM triage cycle, such as those that occurred at 11:00 PM, were not reassigned until the next day’s triage assessment. The model did not explicitly distinguish extubations driven by physiologic improvement from palliative extubations or other human-driven end-of-life decisions, including changes in advance directives or transitions to comfort-focused care; such events were retained only as part of the observed clinical trajectory. ECMO patients were excluded thereafter due to: (1) absence of explicit ECMO considerations in existing CSC protocols, (2) potential misrepresentation of respiratory illness severity by SOFA pulmonary subscores on ECMO, and (3) institutional variability in extubation practices while patients are on ECMO [22]. Because extubation documentation was inconsistent, simulations used a daily time-step for all strategies.

### SOFA scores and imputation

SOFA scores were obtained from the health record for each ventilated day, with preference for data closest to 10:00 AM as possible. Missing pulmonary SOFA subscores were substituted with mSOFA pulmonary scores if available [23, 24]. However, if any SOFA subscore remained missing, it was assumed normal (score of 0), consistent with prior simulation studies, and consistent pragmatically with what occurs in reality [23–26]. If no SOFA score was documented on the day of consideration for intubation, the next day’s SOFA score was used instead for the day of consideration for intubation and any relevant comparisons [22]. 

### Comorbidity and special population assessment

Several ventilator allocation strategies incorporate comorbidities or special populations. The simulation dataset combined electronically extracted structured clinical data with targeted manual chart review for variables requiring strategy-specific adjudication (Supplemental Table 1). Variables requiring manual review were performed by BCW and supervised by DP. Structured data, including demographics, ventilator use, and SOFA-related variables, were extracted electronically from the health record. Comorbidities and strategy characteristics were initially identified through electronic ICD-10 code queries, after which manual chart review to determine whether each condition met the strategy-specific severity thresholds documented by different ventilator allocation strategies and shown in Table 2. Occupation (Table 3) for CAN, PA, and UC strategies was electronically queried and manually extracted. Demographics, including age, sex, and address, were electronically extracted. A detailed comparison of how NY, iNY, CAN, MD, PA, and UC ventilator strategies were simulated is summarized in Table 4.

**Table 2:** Criteria for Simulated Ventilator Rationing Strategies

	RationingStrategy	Strategy Impact	Criteria	Operationalized in Simulation
Cardiac Arrest	NY	Exclusion	Cardiac arrest: unwitnessed arrest, recurrent arrest without hemodynamic stability, arrest unresponsive to standard interventions and measures; trauma	
	MD	Exclusion	Cardiac arrest: unwitnessed, recurrent, or unresponsive to defibrillation or pacing	
	CAN	Exclusion	Cardiac arrest, regardless of age, with 1 of the following poor prognostic factors; unwitnessed cardiac arrest, PEA, recurrent cardiac arrest	
	UC	Exclusion	Unwitnessed out of hospital cardiac arrest without ROSC prior to arrival; any witnessed cardiac arrest with inability to obtain ROSC after 60 min from onset without a shockable rhythm present	
	UC	Exclusion	Coma (inability to respond to verbal commands) after ROSC from cardiac arrest with non-shockable rhythm without confounding drugs, toxins, or metabolic derangements	
Traumatic Brain Injury	NY	Exclusion	Traumatic brain injury with no motor response to painful stimulus (i.e., best motor response = 1) [no motor response to painful stimulus]	
	CAN	Exclusion	TBI meeting all of the following; age > 60, GCS < 8, 1 or both unreactive pupils	
	UC	Exclusion	Traumatic brain injury with >= 90% predicted death on IMPACT score	
Trauma	MD	Severe	Severe trauma	Operationalized as trauma injury severity score predicting >= 90% mortality
	CAN	Exclusion	Trauma with ISS > 16, unless determined to be acutely reversible	
	UC	Exclusion	Trauma injury severity score predicting >= 90% mortality	
Neurological	MD	Exclusion	Advanced and irreversible neurologic event or condition	Queried Dementia (MOCA < 10, MMSE < 10, Clinical Frailty Score ≥ 8), Alzheimer’s (MOCA < 10, MMSE < 10, Clinical Frailty Score ≥ 8), Parkinson’s (MOCA < 10, MMSE < 10, Clinical Frailty Score ≥ 8), Huntington’s, Coma (in persistent vegetative state), or documentation of “advanced and irreversible neurological event or condition”
	MD	Severe	Advanced untreatable neuromuscular disease	ALS with documented “advanced”
	CAN	Exclusion	Severe, irreversible and terminal neurologic event or condition (end-stage dementia)	Dementia & Clinical frailty score ≥ 8 or Rankin score ≥ 4
	CAN	Exclusion	Advanced untreatable neurodegenerative disease (ALS, Parkinson Disease)	ALS (documented “advanced”), Parkinson’s (Clinical frailty score ≥ 8 or Rankin score ≥ 4)
	PA	Severe	Severe Alzheimer’s disease or related dementia	MOCA < 10, MMSE < 10, or explicitly written in chart
	UC	Major	Pre-existing neurological condition (dementia, stroke, other neurodegenerative disease) with baseline modified Rankin Score > 4	Dementia, CVA
	UC	Severe	Minimally conscious or unresponsive wakeful state from prior neurological injury	Coma (in persistent vegetative state), Alzheimer, or Parkinson’s
Stroke	CAN	Exclusion	SAH with WFNS grade V	On admission
	CAN	Exclusion	CVA with age > 70 with large MCA territory, substantial deficits, not amenable to reperfusion	
	CAN	Exclusion	Posterior circulation stroke with GCS < 8	
	UC	Major	Non-traumatic intracerebral hemorrhage with max ich score >9	
	UC	Major	Aneurysmal subarachnoid hemorrhage with HAIR score =8	
	UC	Major	Coma in ischemic stroke with brainstem infarction due to basilar artery occlusion which is non-revascularized or without clinical improvement after revascularization	
Burns	NY	Exclusion	Severe burns: where predicted survival ≤ 10% even with unlimited aggressive therapy; age 50 & 80% survival; age 60 & 70% survival; age 70 & 50% survival	On admission
	MD	Exclusion	Severe burns in patient with both of the following: age 60+ & 50% of total body surface area affected	
	CAN	Exclusion	Burns with 2 of the following: age > 60, > 40% BSA, inhalational injury	
	UC	Exclusion	American burn association expected mortality ≥ 90% age 50 & 80% survival; age 60 & 70% survival; age 70 & 50% survival	
Malignancy	MD	Severe	Metastatic malignant disease or high-grade primary brain tumors	Stage IV or high-grade primary brain tumor
	PA	Severe	Cancer being treated with only palliative interventions (including palliative chemotherapy or radiation)	Explicitly documented palliative intent of cancer treatment, or receiving palliative radiation therapy
	CAN	Exclusion	metastatic malignant disease with expected month of survival < 6mo	Metastatic cancer and documented qualified for hospice
	UC	Severe	Metastatic cancer with expected survival ≤1 year despite treatment	Unable to operationalize
	UC	Severe	Refractory hematologic malignancy (resistant or progressive despite conventional initial therapy)	Documented treatment refractory or progression of disease
	UC	Severe	Terminal illness with Clinical Frailty Scale Score ≥8	Malignancy & clinical frailty score ≥ 8
Cardiovascular	MD	Severe	New York Heart Association class IV heart failure	
	PA	Severe	New York Heart Association Class IV heart failure plus evidence of frailty	Frailty defined as clinical frailty score ≥ 6
	CAN	Exclusion	NYHA class III or IV	
	UC	Major	ACC/AHA Stage C heart failure, NYHA Class II-IV	
	UC	Major	Severe, inoperable multi-vessel coronary artery disease or valvular disease	
	UC	Severe	ACC/AHA Stage D heart failure	
Pulmonary				
COPD	MD	Severe	Advanced lung disease with FEV1 < 25% predicted, total lung capacity < 60% predicted, or baseline Pao2 < 55 mm Hg	In diagnosis of COPD, pulmonary fibrosis, pulmonary hypertension, or cystic fibrosis
	PA	Severe	Severe chronic lung disease plus evidence of frailty	FVC ≤ 35%, FEV1 ≤ 50%, clinical frailty score ≥ 5
	CAN	Exclusion	COPD w FEV1 < 30% or baseline pa02 < 55, or secondary pulmonary hypertension	
	UC	Major	Moderately severe chronic lung disease (e.g., COPD, IPF) but not requiring chronic oxygen or ventilation	GOLD criteria: moderate: 50% ≤ FEV1 <80% predicted
	UC	Severe	Severe chronic lung disease with FEV1 < 20% predicted, FVC < 35% predicted, or in absence of PFTs, chronic home O2 at rest or mechanical ventilation	
Pulmonary Hypertension	MD	Severe	Primary pulmonary hypertension with NYHA class III or IV heart failure	
	CAN	Exclusion	Pulmonary hypertension with NYHA class IV symptoms	
	UC	Major	WHO Class 3 pulmonary hypertension (symptomatic with minimal exertion, asymptomatic only at rest)	
	UC	Severe	WHO Class 4 pulmonary hypertension	
Other	CAN	Exclusion	Cystic Fibrosis with postbronchodilator fev1 < 30% predicted or baseline pa02 < 55mmhg	
	CAN	Exclusion	Pulmonary fibrosis with vital capacity or total lung capacity < 60% predicted, baseline pa02 < 55mmhg or secondary pulmonary hypertension	
Cirrhosis	MD	Severe	Chronic liver disease with Child-Pugh score > 7	
	PA	Severe	Cirrhosis with MELD score ≥20, ineligible for transplant	
	CAN	Exclusion	Cirrhosis with MELD > 20	
	UC	Major	Cirrhosis with MELD <20 and history of prior decompensation	
	UC	Severe	Cirrhosis with MELD score ≥20	
Renal	PA	Severe	End-stage renal disease in patients older than 75	
	UC	Major	End stage renal disease on dialysis	
Other	CAN	Exclusion	advanced and irreversible immunocompromise	Unable to operationalize
	NY	Exclusion	Irreversible age-- related arrest specific hypotension unresponsive to fluid resuscitation and vasopressor therapy	Unable to operationalize
	NY	Exclusion	Any other conditions resulting in immediate or near immediate mortality even with aggressive therapy (This “catch all” phrase encompasses other possibilities because the list above is merely a guide and does not list every medical condition that would result in immediate or near-immediate mortality.)	Unable to operationalize
	CAN	Exclusion	Age 80+ and clinical frailty score ≥ 5	Queried chart for clinical frailty score, rankin score, ECOG, surprise question, karnofsky score, ADLS, iADLs in every chart of an individual aged 80+
	CAN	Exclusion	Advanced and irreversible immunocompromise	Unable to operationalize

Strategy impact: Table terminology was harmonized across strategies for readability. Severe and Major comorbidity modifiers deprioritized patients by adding +4 and +2 points, respectively, to the strategy-specific triage score. Patients meeting Exclusion criteria were assigned to the lowest triage priority category and received a ventilator only if a surplus remained after allocation to all higher-priority groups

**Table 3:** Occupation Criteria for Triage Prioritization

Canada	Pennsylvania	University of California
Healthcare Occupation	Essential Occupation	Critical Occupation
Health care workers have instrumental value (i.e., a health care worker who is healthy can save the lives of more patients). However, during a stage of critical illness, it is unclear whether the health care worker, if saved, would be able to return to work in a timely fashion to help others. Instrumental value is a potentially subjective concept that lends itself too easily to other, potentially extraneous, considerations of social worth. Instrumental value (prioritize those who have particular instrumental value to others in the pandemic, e.g., health care workers).	Rather than extending this priority only to frontline clinicians, it should be given to all workers in essential jobs that face high risk of infection because of frequent workplace exposures, such as grocery store workers, bus drivers, home health workers, and food service workers.	Occupation, EMTs, police officers, firefighters, clinical healthcare workers, [paramedic, medic] (any health care worker who (a) has been disproportionately exposed to COVID-19 through the workplace, and who works in a field that is (b) necessary for the control of the pandemic, and where in addition (c) prioritizing workers in that field is reasonably viewed as a necessary step to maintain adequate medical staffing in that field during the pandemic.), other individuals who, over time, might be revealed to have a multiplier effect roughly equivalent to the individuals above, given the specific way the pandemic unfolds.

**Table 4:** Detailed Comparison of Criteria used in Simulated Ventilator Rationing Strategies

		New York (NY / iNY *)				Canada (CAN)		Maryland (MD)		Pennsylvania (PA)		University of California (UC)				
Assessment & Reassessment (days of ventilation)		D_0_	D_2_	D_5_	D_+ 2_	D_0_, D_+ 3_		D_0_, D_1_, D_2_, D_5_, D_+ 2_		D_0_, D_+ 3_		D_0_, D_+ 3_ **				
SOFA	1 point	≤ 7	↓ & ≤ 11	≤ 7 Δ ≥ 3		≤ 7		≤ 8		< 6		< 6				
	2 points	8–11	ØΔ ≤ 7	≤ 7 Δ ≤ 3	↓ ≤ 7	> 7		9–11		6–8		6–9				
	3 points	> 11	> 11 ØΔ 8–11 ↑	> 7 Ø Δ ≤ 7 ↑	> 7 Ø Δ ↑			12–14		9–11		10–12				
	4 points							> 14		≥ 12		> 12				
Modifications								De-Prioritize	Severe Comorbidities + 4	De-Prioritize	Severe Comorbidities + 4	De-Prioritize	Severe Comorbidities + 4			
												De-Prioritize	Major Comorbidities + 2			
												Prioritize	Pregnancy − 2			
												exempt	Critical Care Worker (exempt 3 days, then -4 at day 3, then − 2 indefinitely) ***			
								De-Prioritize	Unchanged SOFA on days 0 to 5; +1	Prioritize	Structural Inequalities (ADI) -1	exempt	Non-Transplant Post-Operative Complex Surgery, exempt for 5 days			
								Prioritize	Pregnancy − 1			exempt	Post-Operative Transplant, exempt for 10 days			
Group Prioritization (sum of point-modifiers, exclusionary criteria)		*Sequentially* 1. In-between Reassessment 2. 1 Point 3. 2 Points 4. 3 Points 5. Exclusion Criteria				*Sequentially* 1. In-between Reassessment 2. 1 Point 3. 2 Points 4. Exclusion Criteria		*Sequentially* 1. In-between Reassessment 2. 1 Point 3. 2 Points 4. 3 Points 5. 4 Points 6. 5 Points 7. 6 Points 8. 7 Points 9. 8 Points 10. 9 Points 11. Exclusion Criteria		*Sequentially* 1. In-between Reassessment 2. -1 Point 3. 0 Points 4. 1 Point 5. 2 Points 6. 3 Points 7. 4 Points 8. 5 Points 9. 6 Points 10. 7 Points 11. 8 Points				Days from start of crisis		
														**D**_**0**_	**D**_**3**_	**D**_**+ 3**_
												**Priority** **1st**		Exempt		
												**Priority** **2nd**		*po* *ints* -1 to 3	*points* -5 to 3	*points* -2 to 3
												**Priority** **3rd**		*points* 4–6	*points* 4–6	*points* 4–6
												**Priority** **4th**		*points* 7–8	*points* 7–8 Δ ≥ 3	*points* 7–8 Δ ≥ 2
												**Priority** **5th**		Exclusionary Criteria		
Tie Breaker		Randomization				1st	If Excluded, SOFA ≤ 7	1st	Age Goup 1) < 50 2) 50 to 70 3) 71 to 85 4) > 85	1st	Age Goup 1) < 40 2) 40 to 60 3) 61 to 75 4) > 75	Randomization				
						2nd	Pregnancy									
						3rd	Healthcare Worker ***									
						4th	Age Goup 1) < 40 2) 40 to 60 3) 61 to 75 4) > 75	2nd	Randomization	2nd	Randomization					
						5th	Randomization									

* iNY further subcategories the Blue category into, in descending priority, those with a SOFA score on the day of evaluation of (i) ≤ 7, (ii) 8–11, or (iii) > 11

** California: Ventilator rationing occurs every day but triage group prioritization are only determined every 3 days

*** See Table 3

### Ventilator allocation strategies

Each strategy simulated how ventilators would be allocated under defined CSC policies. Below is a brief summary of the operational details of ventilator rationing for each policy specifically during the crisis period. Each strategy can be accessed at https://github.com/bcorbettwalsh/CSC/tree/main.

### Lottery system strategy

Each day during the crisis period, ventilators were randomly assigned to all patients requiring one until the supply of ventilators was exhausted.

### First-Come First-Served (FCFS) strategy

Ventilators were allocated sequentially based on the time of intubation. Because FCFS inherently depends on preserving the temporal order of mechanical ventilation rather than standardized daily assessment, original intubation times were retained and newly available ventilators were assigned beginning at midnight. Patients assigned a ventilator retained it until extubation, death, or ECMO initiation.

### New York ventilator allocation guidelines 2015 (NY [14] & iNY [4])

As previously described [4], New York (NY) & improved New York (iNY) guidelines followed three steps: Step 1–application of exclusionary criteria; Step 2–consideration of providing a ventilator; and Step 3–potential ventilator reallocation after a pre-specified time trial. Under NY guidelines, patients are prioritized to receive or maintain a ventilator as High, Intermediate, or Low (only provided a ventilator if a surplus exists), based on either a single SOFA score (static on day of intubation), or a SOFA trend (dynamic comparison of the SOFA score on the day of assessment to the previous SOFA assessment). Triage assessments were performed on the day of intubation, day 2, day 5, and every 2 days of ventilator use thereafter. On any day during the simulated crisis period, ventilators were first provided to those individuals not due to be reassessed (i.e., those that already were receiving a ventilatory time trial), then randomly to patients categorized as High, then randomly to those patients classified as Intermediate, and lastly randomly to patients triaged as Low. If ventilator demand exceeded supply within a priority group, allocation was randomized for the group.

An improvement to NY (iNY) subcategorized the Low category group based on acuity of illness on the day of evaluation. Ventilators would be rationed away sequentially as follows: first from individuals categorized as Low whose SOFA score was > 11 after already receiving a time trial, then from Low individuals considered for intubation but with a SOFA > 11 or those with a SOFA score between 8 and 11 after a time trial, and finally from those Low individuals with a SOFA < 7 after a time trial.

### Saskatchewan, Canada 2020 guidelines (CAN) [10]

Ventilators were allocated first to those not due for reassessment, followed by those with a SOFA score ≤ 7, then to those with a SOFA score > 7, and finally to patients who met exclusionary criteria. Within each of these priority groups, ties were resolved using the following secondary criteria, in order: pregnancy, occupation (see Table 3), age group (prioritized in order of age less than 40, then 41–60, 61–75, and then greater than 75 years), and finally randomization. For patients in the exclusionary category, further prioritization was based on SOFA score (≤ 7 before > 7) before applying the same secondary criteria. Triage assessment was performed on the day of intubation and every 3 days of ventilator use thereafter. Modification to the original published strategy included subgrouping those satisfying exclusionary criteria into those with a SOFA score ≤ 7 and those with a SOFA score > 7.

### Biddison et al. 2019 Maryland guidelines (MD) [11]

Patients were prioritized to receive or maintain a ventilator based on their total triage priority score, composed of a severity of illness score and comorbidity elements. A patients severity of illness was determined by the SOFA score on the day of evaluation (1 point: SOFA ≤ 8; 2 points: SOFA 9–11; 3 points: SOFA 12–14, 4 points: SOFA > 14) and a comparison modifier (+ 1 if SOFA score was worsening between SOFA reassessment scores or + 1 if SOFA remained unchanged between day of intubation and day 5 reassessment). The total priority score was further modified by the presence of a severe comorbidity (+ 4). Ventilators were provided first to those individuals not due to be reassessed, then sequentially to total triage priority scores 1–9, and then to those satisfying exclusionary criteria. Ties within each category were resolved by age group (age less than 50, then 50–70, 71–85, and then greater than 85) and then by randomization. Triage assessment was performed on the day of intubation, day 1, day 2, day 5, and every subsequent 2 days (subsequent evaluation was not originally included, so 2 days was chosen). A 2021 published iteration [27] utilized the same SOFA criteria, but made slight revisions: replaced the comorbidity modifier with “severe comorbid conditions with death likely within 1 year” (+ 3), pregnancy modifier (-1); ties were resolved by prioritizing “individuals who are improving the most” and then randomization; and triage assessments were increased to daily. The pregnancy modifier (-1) was included in the model, but further adjustments were not made as they could not be operationalized.

### University of Pittsburgh 2020-2021 guidelines (PA) [9, 17]

Patients were prioritized to receive or maintain a ventilator based on their total triage priority score, composed of a severity of illness score and comorbidity elements. A patient’s severity of illness was determined by their SOFA score on the day of intubation (1 point: SOFA < 6; 2 points: SOFA 6–8; 3 points: SOFA 9–11, 4 points: SOFA > 11). The total priority score was further modified by the presence of a severe comorbidity (+ 4), occupation as an essential worker (-1, Table 3), or to address structural inequities (-1; analysis includes Area Deprivation Index, ADI, score of 8 or more unless otherwise stated. The Social Vulnerability Index, SVI, of greater than 7500 was used in a separate analysis). Ventilators were provided first to those individuals not due to be reassessed, then sequentially to ascending total triage priority scores from − 1 to 8. Ties within each category were resolved by age group (age less than 40, then 40–60, 61–75, and then greater than 75 years) and then by randomization. Triage assessment was performed on the day of intubation and every 3 days of ventilator use thereafter. The simulated PA guideline is modified from the original 2020 version (triage occurs every 3 days, removal of moderate comorbidities). The model includes elements from both the 2020 and the subsequent 2021 iterations including structural inequalities, but not the replacement of SOFA scores and comorbidities with mortality estimates, as these could not be operationalized.

### University of California 2020 guidelines (UC) [16]

Patients were prioritized to receive or maintain a ventilator based on their total triage priority score, composed of a severity of illness score and comorbidity elements. A patient’s severity of illness was determined by the SOFA score on the day of evaluation (1 point: SOFA < 6; 2 points: SOFA 6–9; 3 points: SOFA 10–12, 4 points: SOFA > 12). The total priority score was further modified by the presence of a severe comorbidity (+ 4), a major comorbidity (+ 2), or if they were pregnant (-2). Those with an occupation as a critical care worker were exempt from triage for 3 days, then received a -4 modifier for 3 days, and then a -2 modifier indefinitely. Patients who were post-operative of a transplant complex surgery were exempt from triage for 10 days from the date of surgery and subsequently triaged without adjustment. Patients who were post-operative of a non-transplant complex surgery were exempt from triage for 5 days from the date of surgery and subsequently triaged without adjustment. Ventilators were assigned each day; however, their triage priority was only determined at the start of the crisis period and every subsequent 3rd day (or the day of intubation if patients were intubated after the start of the crisis period). Ventilator allocation proceeded first to those exempt from ventilator triage, then to those with a total priority score ≤ 3, then those with a total priority score between 4 and 6, then those with a total priority score ≥ 7 or if their overall priority score increased by 3 or more points on the 3rd day of the crisis compared to the first, or if it increased by 2 or more points on any subsequent day of evaluation compared to the prior day of evaluation. Any surplus of ventilators would be assigned to individuals meeting exclusionary criteria. Ties within each priority category were resolved by randomization.

### Statistical analysis

Outcome endpoints include both observed (real-world) and simulated (hypothetically in the simulation). The primary observed endpoint was survival to hospital discharge, which is known because every patient in our cohort received a ventilator in the real world. The primary simulated endpoint was the overall crisis cohort survival under each strategy. Secondary simulated endpoints included the number of patients denied or removed from ventilators during the simulation, and the observed mortality for those individuals selected for ventilator rationing.

Additional secondary analyses focused on evaluating social disparities among those whose ventilator was rationed including patient demographics: age, sex, race and ethnicity, neighborhood-level social vulnerability (measured using ADI or SVI), and comorbidities. We also reviewed the day of ventilation when rationing occurred, triage priority category, and whether the patient met any strategy-specific special criteria (e.g., pregnancy, occupation).

Tertiary simulated endpoints included, for each patient selected for ventilator rationing in a single simulation, the frequency with which they were selected across all simulations.

### Simulation and statistical tools

Each ventilator allocation strategy was simulated 10,000 times using R (R Core Team 4.3.1, 2021, Vienna, Austria). Simulated endpoint results were averaged across the 95% of simulations with the shortest crisis period duration for each strategy. Categorical data was compared using the chi-square test, with statistical significance set at *p* < 0.05. Continuous variables were analyzed with a two-tailed t-test using a 5% significance level. Analysis was completed in R and Excel 2022 (Microsoft). This study followed the Strengthening and Reporting of Observational Studies in Epidemiology (STROBE) reporting guideline, was approved by NYU Grossman School of Medicine’s IRB (i20-01653) with a waiver of informed consent, and was funded by a CHEST Foundation COVID-19 Research Grant.

## Results

The surge cohort included 2,365 mechanically ventilated patients, and simulation models have been completed for all eight CSC strategies. Post-simulation analyses will be published separately. The predefined crisis threshold–95% pre-pandemic ventilator usage triggering CSC rationing–corresponded to 71.8% of the observed peak ventilator use during the actual surge. This level of operational strain is consistent with thresholds simulated in prior CSC simulation studies [28]. 

## Discussion

Simulating CSC during prior COVID-19 surges offers an ethically sound and logistically feasible method for evaluating the performance of ventilator triage strategies. Retrospective simulations during periods of profound but non-crisis resource strain–when care was almost but not formally rationed–offer a unique methodological advantage: because all patients received life-saving interventions, we can directly observe the outcomes of individuals who, under CSC protocols, might have been denied such life-sustaining resources. This enables estimation of counterfactual mortality across different triage strategies using real-world survival data. Moreover, retrospective modeling facilitates an estimation of resource allocation efficiency and equity without the ethical hazards of real-world, real-time experimentation.

Unfortunately, with few exceptions [23, 28], most CSC simulations have focused exclusively on initial triage decisions, omitting reassessment or reallocation decisions [15, 20, 25, 29, 30]. While this approach simplifies simulation modeling, it fails to capture the temporal complexity of real-world resource strain. Failure to incorporate dynamic resource strain over time can obscure the actual duration and intensity of the crisis period. This risks including patients whose care occurred outside the intended simulated crisis window, potentially misattributing outcomes and biasing strategy performance [19]. In addition, excluding reassessment triage ignores a fundamental component of nearly all CSC protocols, namely the requirement for ongoing periodic re-evaluation of benefit. Failure to model this process may substantially misrepresent how CSC policies would function under real-world surge conditions.

This framework should also be interpreted in light of several limitations. First, although this design avoids the unrealistic assumption used in many bootstrapped simulations of one-time triage decisions, namely that smaller patient groups can be randomly sampled from a larger cohort and subjected to an arbitrary level of resource scarcity as though all patients presented simultaneously [15, 20, 25, 31], it still simplifies ventilator rationing into discrete daily assessment points, which does not fully reflect real-world surge dynamics [32]. While this pragmatic modeling choice may have introduced temporal imprecision relative to real-world practice, it may better approximate how triage committees operate by making allocation decisions at defined clinical intervals. Second, because this was a retrospective simulation based on observed clinical trajectories, the model remained susceptible to information bias and possible misclassification, particularly for variables requiring manual adjudication. Third, missing SOFA subscores or total scores were assumed to be normal when no data were otherwise available, which may have introduced optimistic or pessimistic bias depending on initial or reassessment decision nodes or the strategy modeled [33]. Fourth, this retrospective simulation relied on observed clinical trajectories and did not explicitly model dynamic changes in goals of care, advance directive revisions, or terminal extubation decisions. This limitation may affect estimates of ventilator duration and availability, as palliative extubations may not reflect physiologic recovery and could influence downstream allocation dynamics. Finally, the patient cohort was derived from a single academic health system during the initial COVID-19 surge in New York City, and model performance may differ in settings with different patient populations, documentation practices, resource constraints, or institutional workflows.

The inclusion of admission diagnosis and existing comorbidities into CSC triage has raised concerns among disability advocates [34, 35], prompting a formal response from the U.S. Department of Health and Human Services Office of Civil Rights (HHS-OCR) [36]. In 2022, HHS-OCR issued updated guidance [37] that led several states to revise their CSC protocols to comply with existing federal nondiscrimination regulations [34]. Many of the comorbidity-based criteria used in the strategies simulated in this study may raise similar legal or ethical objections of discrimination, particularly when allocation decisions are not based on individualized survival predictions prior to hospital discharge [38]. Age, a commonly debated triage factor, was included in nearly all strategies simulated in this study–either explicitly (CAN, NY, UC, and PA strategies) or as a part of tiebreaker criteria (CAN, MD, and PA strategies). While HHS-OCR has provided guidance on how age may be legally and ethically incorporated into CSC frameworks [38], its use remains controversial and requires careful justification.

## Conclusions

We developed a high-fidelity, daily time-stepped simulation platform to compare multiple real-world crisis standards of care ventilator allocation strategies during a COVID-19 surge. This methods-focused manuscript provides a detailed account of how diverse triage strategies–including their dynamic reassessment rules, comorbidity and triage modifier criteria, and operational constraints–were rigorously implemented and evaluated using retrospective data from a large New York City health system. This simulation framework advances prior models by incorporating realistic patient throughput, time-dependent reassessment, and strategy-specific allocation rules. By leveraging observed outcomes from a period of severe but non-crisis strain, we were able to simulate counterfactual survival outcomes across alternative allocation strategies while avoiding the ethical and logistical challenges of real-world CSC testing. The methods presented here offer a detailed, transparent, and reproducible simulation foundation, which can be readily adapted for broader ethical, policy, and operational evaluation of CSC strategies in other contexts. While this report focuses on model construction and methodological detail, simulation outcomes–including survival rates, rationing efficiency, and equity metrics–will be reported separately. Future research may extend this platform to evaluate additional triage frameworks and support the development of more equitable and effective resource allocation strategies for future disaster preparedness and public health response initiatives.

## Supplementary Information

Supplementary Material 1.

## Data Availability

The datasets used and/or analyzed during the current study are available from the corresponding author on reasonable request.
