# Supplementary material for: Simulating crisis triage: a methodological framework for evaluating ventilator allocation under crisis standards of care
Source: BMC Med Res Methodol. 2026 May 26;26:134. doi: 10.1186/s12874-026-02878-1 (PMC13267345; doi:10.1186/s12874-026-02878-1)
Supplement: Supplementary file 1 — Supplementary Material 1. [file 12874_2026_2878_MOESM1_ESM.docx]

| Variable | | Electronically Abstracted | Manually reviewed & verified | Supervised |
| --- | --- | --- | --- | --- |
| Obtained Daily | PaO2 | + |  |  |
|  | SpO2 | + |  |  |
|  | Fi02 | + |  |  |
|  | Platelets | + |  |  |
|  | Bilirubin | + |  |  |
|  | Mean Arterial Pressure | + |  |  |
|  | Dobutamine | + |  |  |
|  | Dopamine | + |  |  |
|  | Norepinephrine | + |  |  |
|  | Epinephrine | + |  |  |
|  | GCS | + |  |  |
|  | Creatine | + |  |  |
|  | Urine output (24 hours) | + |  |  |
|  | Dialysis | + |  |  |
|  | Presence of ventilator | + |  |  |
|  | Presence of paralytics | + |  |  |
| Obtained per Hospitalization | Pregnant | + | + | + |
|  | Tocilizumab | + |  |  |
|  | Antibiotics | + |  |  |
|  | Steroids | + |  |  |
|  | Remdesivir | + |  |  |
|  | lopinavir / ritonavir | + |  |  |
|  | Azithromycin | + |  |  |
|  | Plaquenil | + |  |  |
|  | Intubation date | + |  |  |
|  | Extubation date | + |  |  |
|  | Admission date | + |  |  |
|  | Discharge date | + |  |  |
|  | Date of death | + |  |  |
|  | ECMO initiation Date | + |  |  |
|  | ECMO de-escalation Date | + |  |  |
| Obtained per Patient | Date of birth | + |  |  |
|  | Sex | + |  |  |
|  | Race | + | + | + |
|  | Ethnicity | + | + | + |
|  | BMI | + |  |  |
|  | Dementia | + | + | + |
|  | Alzheimer | + | + | + |
|  | Stroke | + | + | + |
|  | ALS | + | + | + |
|  | Huntington’s disease | + | + | + |
|  | Parkinsons disease | + | + | + |
|  | Multiple sclerosis | + | + | + |
|  | Coma | + | + | + |
|  | Quadriplegic | + | + | + |
|  | Trauma (admission) | + | + | + |
|  | Traumatic brain injury (admission) | + | + | + |
|  | Subarachnoid hemorrhage (admission) | + | + | + |
|  | Stroke (admission) | + | + | + |
|  | Heart failure | + | + | + |
|  | Coronary artery disease | + | + | + |
|  | Cardiac arrest (admission) | + | + | + |
|  | Pulmonary function tests | + | + | + |
|  | Asthma / chronic obstructive lung disease | + | + | + |
|  | Interstitial lung disease | + | + | + |
|  | Pulmonary hypertension | + | + | + |
|  | Cystic fibrosis | + | + | + |
|  | Chronic kidney disease stage 5 / dialysis | + | + | + |
|  | 6 minute walk test | + | + | + |
|  | Cirrhosis | + | + | + |
|  | Malignancy | + | + | + |
|  | Frailty | + | + | + |
|  | Occupation | + | + | + |
